# Smoothness correction for better SOFI imaging

**DOI:** 10.1038/s41598-021-87164-4

**Published:** 2021-04-07

**Authors:** Siewert Hugelier, Wim Vandenberg, Tomáš Lukeš, Kristin S. Grußmayer, Paul H. C. Eilers, Peter Dedecker, Cyril Ruckebusch

**Affiliations:** 1 Laboratory for Nanobiology, KU Leuven, 3001 Leuven, Belgium; 2 Laboratory of Nanoscale Biology, École Polytechnique Fédérale de Lausanne, 1015 Lausanne, Switzerland; 3 Grußmayer Lab, Delft University of Technology, 2629 HZ Delft, the Netherlands; 4 Erasmus University Medical Centre, 3015 Rotterdam, the Netherlands; 5 University of Lille, CNRS, UMR 8516, LASIRE, 59000 Lille, France

**Keywords:** Super-resolution microscopy, Nanoscale biophysics, Fluorescence imaging

## Abstract

Sub-diffraction or super-resolution fluorescence imaging allows the visualization of the cellular morphology and interactions at the nanoscale. Statistical analysis methods such as super-resolution optical fluctuation imaging (SOFI) obtain an improved spatial resolution by analyzing fluorophore blinking but can be perturbed by the presence of non-stationary processes such as photodestruction or fluctuations in the illumination. In this work, we propose to use Whittaker smoothing to remove these smooth signal trends and retain only the information associated to independent blinking of the emitters, thus enhancing the SOFI signals. We find that our method works well to correct photodestruction, especially when it occurs quickly. The resulting images show a much higher contrast, strongly suppressed background and a more detailed visualization of cellular structures. Our method is parameter-free and computationally efficient, and can be readily applied on both two-dimensional and three-dimensional data.

## Introduction

Super-resolution fluorescence techniques^1–6^ have become increasingly important to study dynamic interactions in live-cell imaging and applications in large scale systems. Several of these techniques achieve a sub-diffraction spatial resolution by relying on the dynamics of the fluorophores, such as controlled on/off switching between a fluorescent and a non-fluorescent state^7^. Single-molecule localization techniques (SMLM), such as stochastic optical reconstruction microscopy (STORM)^2^ or photo activated localization microscopy (PALM)^3^, acquire large numbers of fluorescence images from the same field-of-view, while exploiting the fluorophore dynamics such that each image contains only a low number of active emitters. Having only a few active probes per image makes it possible to directly determine the spatial coordinates of these probes but limits the temporal resolution as many images must be acquired^8^. High-density localization imaging techniques^9–14^, can increase the temporal resolution, but typically require a trade-off with the obtainable spatial resolution and make the calculation significantly less transparent and/or more computationally expensive.

Another strategy for high-density super-resolution imaging is to use a statistical approach based on signal fluctuations^15–17^. In this work, we focus on super-resolution optical fluctuation imaging (SOFI)^17^. In SOFI, hundreds to thousands of fluorescence images are acquired from a sample labeled with ‘blinking’ fluorophores, and the resulting fluorescence dynamics are analyzed statistically to obtain an image with an improved spatial resolution. It can be used directly or combined with other super-resolution techniques such as image scanning microscopy (SOFISM)^18^. SOFI extracts super-resolution information by calculating (cross-)cumulants of the temporal intensity fluctuations, which can be applied both in two-dimensional^19^ and in three-dimensional visualization^17,20^. Compared to other super-resolution approaches, SOFI is notable for continuing to work at (very) high densities of active emitters, while not imposing restrictions on the brightness of the emitters or the background emission of the sample. Furthermore, the SOFI principle can be described by an analytical model^17,21^ which provides a sound mathematical basis that supports the robustness of SOFI imaging, and we developed an approach to obtain unbiased estimates of the per-pixel reliability of a SOFI image^22^.

However, a key assumption of SOFI is that individual emitters are stationary and show independent fluorescence dynamics. Any process that introduces additional correlations between different emitters can thus lead to distortions of the SOFI image. We analyzed this effect for probe diffusion, where the distortion was found to be essentially negligible in biological samples^23^. However, photodestruction of the fluorophores, which is typically unavoidable in fluorescence imaging, results in a correlated decrease of the fluorescence signal in time. It can give rise to spurious SOFI signals that may even lead to images that appear visually more pleasing, yet do not fully capture the true fluorophore distribution*.* We previously investigated a range of methods to correct for this effect^24^ and identified batch correction as a suitable default strategy. In this approach, SOFI images are independently calculated for smaller subsequences of the entire dataset and then combined to deliver the final image. In practice, this usually requires that the batches are small (tens of images), which complicates quantitative comparison of SOFI images acquired with different batch sizes^7,21^.

An important consideration is that the signal introduced by photodestruction occurs at a different (usually much slower) time scale than that of the desired ‘blinking’ signal, suggesting that a smoothing-based approach may deliver a widely-applicable strategy. To this end, we propose a model-free and computationally efficient method to correct SOFI imaging for photodestruction while avoiding the use of batching. Our method is based on Whittaker smoothing^25,26^, previously used in chemical data analysis (e.g. spectroscopy, chromatography, etc.) to smooth signals^26,27^ or to perform baseline correction^28,29^. In principle, our approach cannot only correct for photodestruction, but can remove any spurious correlations that occur on slower timescales than those associated with the fluorophore blinking dynamics, such as slow illumination effects (due to e.g., laser instability). We demonstrate our method by applying it to two- and three-dimensional SOFI imaging.

## Results and discussion

**Figure 1:**
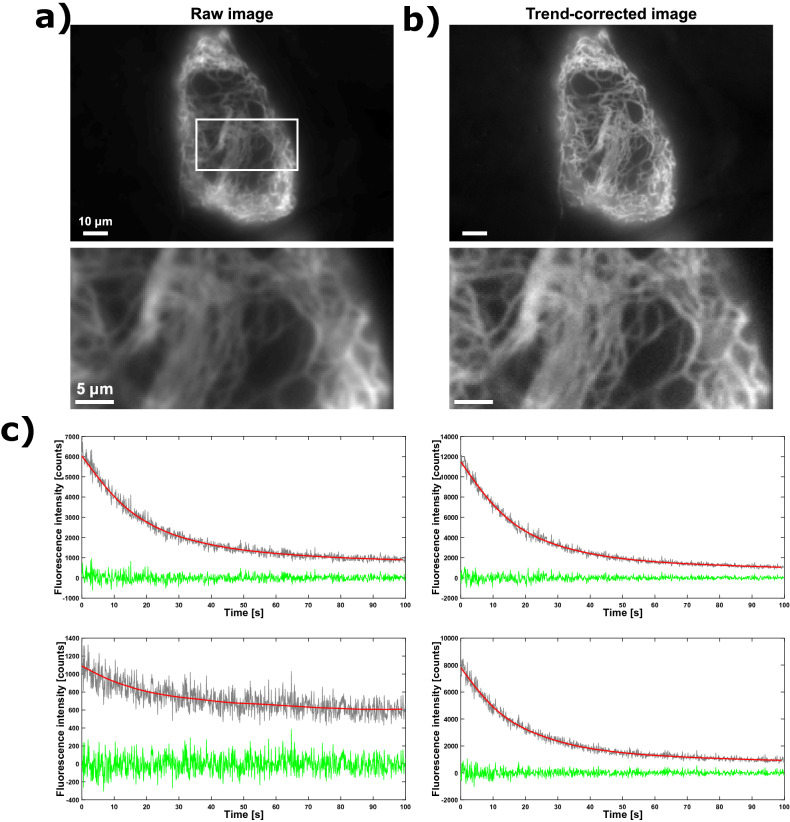
Application of Whittaker smoothing on HeLa cells expressing Vimentin-Dreiklang. The mean image of the raw data and the trend-corrected data are shown in (**a**) and (**b**), respectively. In (**c**), we show four random raw pixel traces (grey curves), their fits (red curves), and trend-corrected data (green curves; obtained by subtracting the red from the grey curves, leaving us only with the fast fluorescence dynamics). Note that an offset has been added to the trend-corrected data (**b**) for visual purposes. All results were obtained using Matlab R2018b (Mathworks, USA).

Figure 1 shows a SOFI dataset acquired on HeLa cells expressing Vimentin-Dreiklang, consisting of 5,000 fluorescence images acquired in rapid succession. The sample shows both clear fluorescence dynamics as well as slower, but pronounced, photodestruction, as is evident from Fig. 1c. We realized that Whittaker smoothing could work well to remove this slower-varying component, owing to its ability to combine global and local properties of the data^30^. The principle of the method is to use penalized least squares to obtain a smooth fit to the data, by simultaneously reducing the error between fit and signal and limiting the variation between subsequent model coefficients.

We independently applied the algorithm to the intensity trajectories associated with each detector pixel (grey lines in Fig. 1c), obtaining smooth curves that reflect only the contribution of photodestruction since fast-changing emitter dynamics are not fitted by this procedure (red lines in Fig. 1c). In its most basic implementation, the algorithm does require the specification of the degree of smoothness by choosing a tunable smoothing parameter $\lambda$, requiring dedicated expertise from the operator. However, we made our analysis parameter-free by automatically determining the value of $\lambda$ (for the image in its entirety) using the V-curve procedure (Supplementary Notes 1 and Supplementary Fig. 1)^31,32^. Subtraction of the smoothed trace from the raw data resulted in an intensity trajectory containing only the fast fluorescence dynamics (the blinking; green line in Fig. 1c). In what follows we will refer to the dataset consisting of only the fast fluorescence dynamics as the ‘trend-corrected data’.

To evaluate the impact of this smoothing on the imaging, we calculated the average fluorescence image for both the unmodified and the trend-corrected data (Fig. 1b). Visual inspection of these images reveals that the removal of the slowly-changing fluorescence signals leads to an increased sharpness in the corrected image, and makes it easier to recognize different structures. This indicates that our approach can accentuate the finer details in the image by removing the broad and indistinct contribution of the photodestruction (see also the zooms in the bottom panel as indicated in the top panel of Fig. 1a).

**Figure 2:**
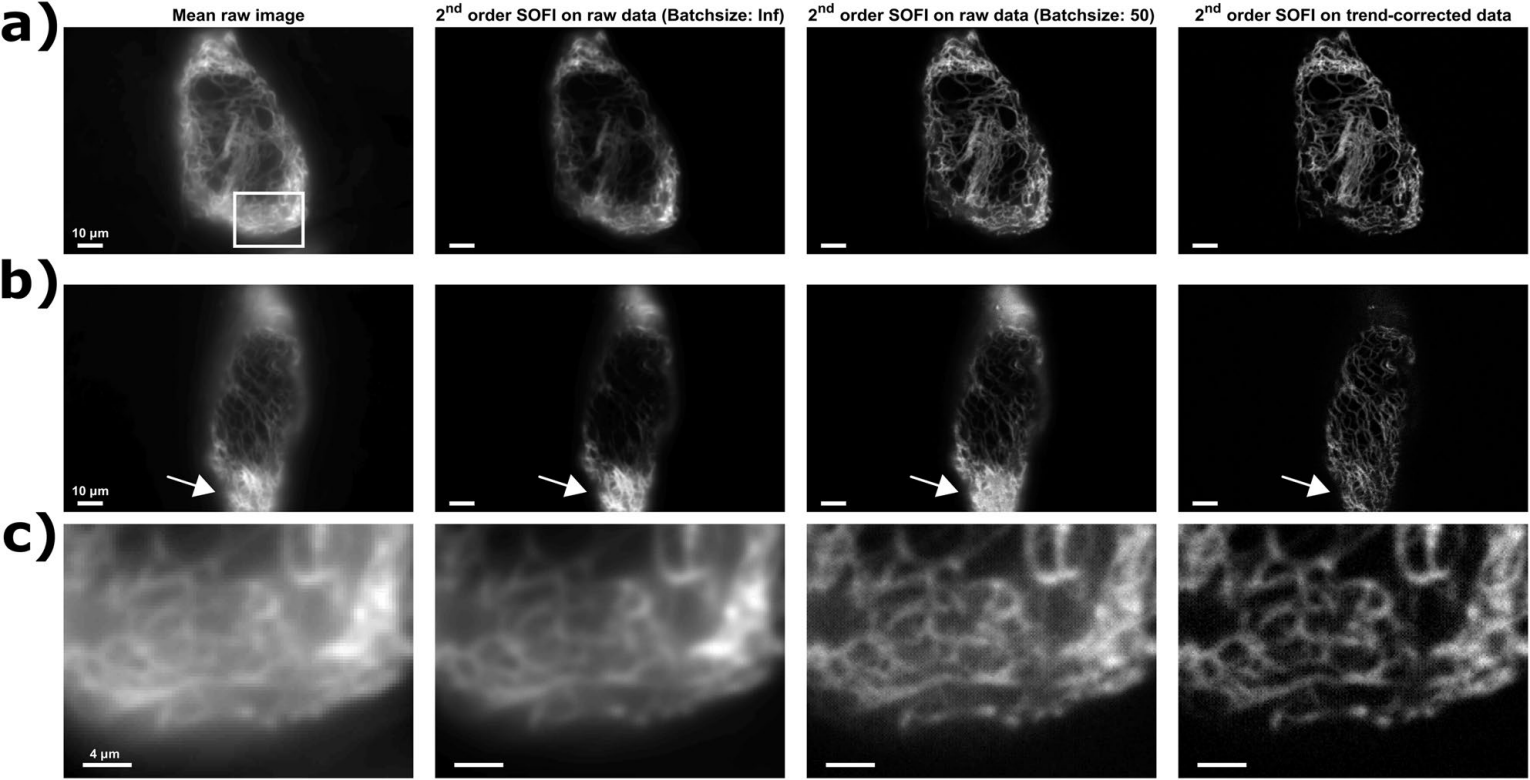
Second order SOFI results on two different datasets as shown in (**a**) and (**b**). The mean raw image is compared to the second order SOFI result on the entire raw data sequence, the raw data using batches of 50 frames and the trend-corrected data. A zoom of the box shown in the left panel of (**a**) is shown in (**c**) where the enhancements after performing the correction method are further highlighted. All results were obtained using Matlab R2018b (Mathworks, USA).

We then applied a second order SOFI analysis to both the raw and the trend-corrected datasets. Figure 2 shows raw SOFI cross-cumulants (with zero time lag), calculated according to^22^ without additional postprocessing for both the raw data and the same data corrected for photodestruction using batching and Whittaker smoothing. As this figure shows, both photodestruction correction strategies resulted in dramatic enhancements over the uncorrected data, which is especially clear in the zoom in Fig. 2c (indicated by the box in Fig. 2a). However, our data also shows a strong gain in detail between the batching-corrected and trend-corrected images, where the latter provides a much clearer visualization of the cellular structure. The overall contrast is also enhanced through improved background reduction (see also Fig. 2c) which leads to an additional discrimination between the structure of interest and the non-specific fluorescence.

Our results show the limitations of using batch correction during SOFI analysis, especially for data showing fast photodestruction, where sharp details can be masked by the only partial suppression of the photodestruction-based signal. This is not the case when using the proposed correction method. Additionally, it is also clear that the SOFI result on the corrected data suffers less from the influence of bright spots present (as for example indicated by the arrow in Fig. 2b), as they are prominently present in the non-corrected and batch corrected SOFI image and mask the details of the filaments, whereas the filament structures can be clearly distinguished in the SOFI image obtained on the trend-corrected data. The reason that the photodestruction signal manifests itself as these bright spots is that photodestruction introduces correlations between neighboring molecules ^24^, which can be augmented by out-of-focus fluorophores or background fluorescence.

The reason why Whittaker smoothing works so well is because it does not rely on an a priori physical model of the behavior of the data, which is especially useful in experimental data as they contain complicated signals that deviate from the ideal situation due to possible interaction between the different fluorophores within the cells, and measurement artefacts (e.g., non-homogeneous illumination, sample scattering, etc.). As all pixels are smoothed individually, these effects, as well as spatial effects, are therefore ignored during the optimization, which leads to smooth fits tailored to different parts of the image. Additionally, there is a clear gap in terms of signal frequency between the smooth photodestruction contributions, or other contributions such as illumination effects, and the high-frequency blinking signals in SOFI measurements, which further facilitates their separation.

**Figure 3:**
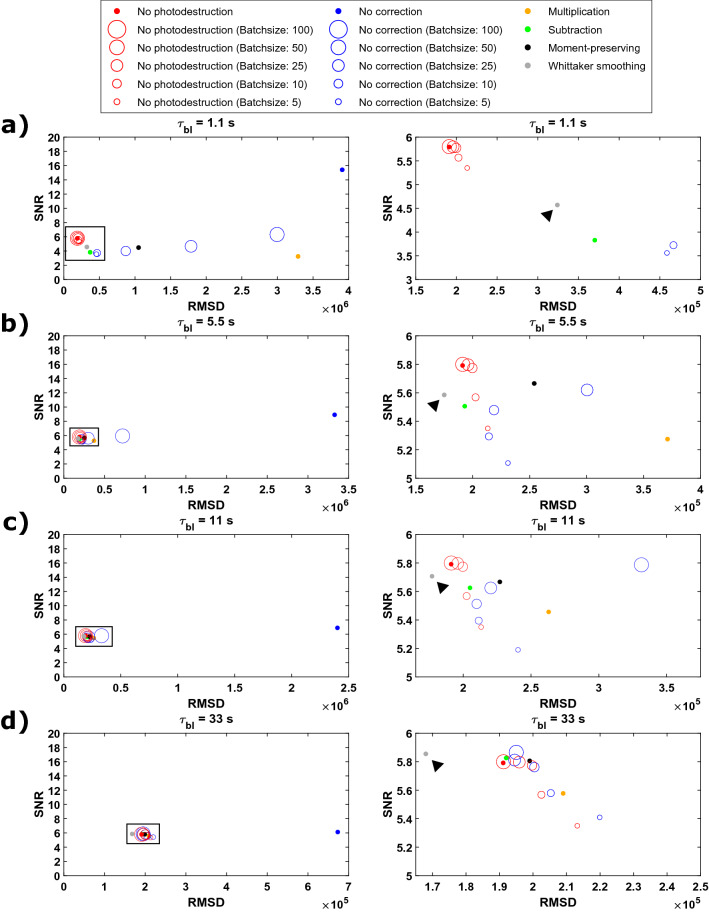
Evaluation of the proposed Whittaker smoothing correction method (indicated with an arrow) in comparison with previous results obtained as reported in previous work^24^. The obtained SNR and RMSD values by using second order SOFI analysis on four different sets of simulations with different photodestruction behavior, with survival times ($\tau_{bl}$) set at 1.1 s, 5.5 s, 11 s and 33 s are shown in panels (**a**) to (**d**), respectively. Correcting photodestruction with Whittaker smoothing gives extremely good results overall, but it especially has a big advantage over the other methods in situations where photodestruction is faster (panels** a**–**b**). All results were obtained using Matlab R2018b (Mathworks, USA).

We then performed a more systematic evaluation of the performance of Whittaker smoothing to correct for photodestruction and contrasted it with existing approaches, analogous to what was done previously^24^. Four different situations (100 repetitions each) with each a different photodestruction behavior were evaluated according to signal-to-noise (SNR) and root mean square deviation (RMSD) metrics (see Methods). The survival times $(\tau_{bl} )$ were set at 1.1 s, 5.5 s, 11 s and 33 s to be able to evaluate the proposed correction method over a broad range of photodestruction rates. The results obtained with the proposed method were combined with the results obtained in the previous work and are shown in Fig. 3 (a zoom of the black rectangle in the left panels is shown in the right panels, where the proposed method is indicated with an arrow). These show that the proposed correction performs equally well in situations where photodestruction is slow with respect to the other methods (Fig. 3c and d) but outperforms all other methods when photodestruction is faster (Fig. 3a and b), with high SNR and low RMSD values as a result. Our methodology using Whittaker smoothing distinguishes itself from other approaches in that it considers the entire intensity trajectory of a pixel at once, readily adapting to local changes by the smoothing penalty in its optimization. A comparison between the SOFI images obtained with different methods on simulations with fast photodestruction can be found in Supplementary Figure 2.

**Figure 4:**
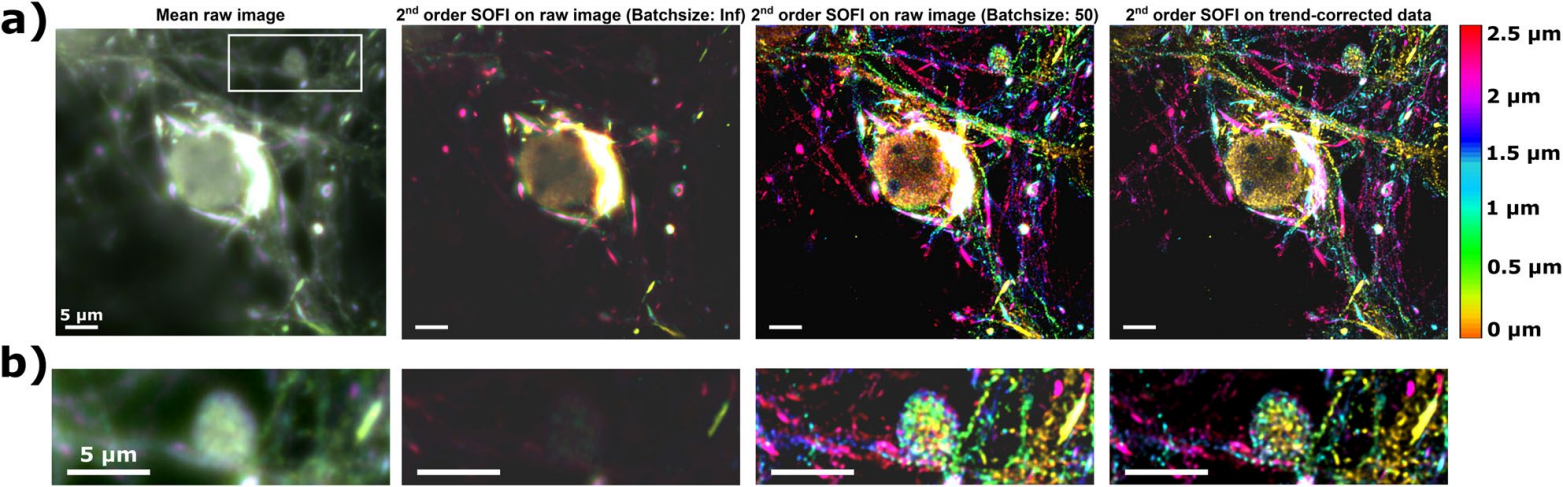
SOFI results on α-synuclein aggregates in a primary hippocampal neuron (treated with α-synuclein pre-formed fibrils and antibody-labeled with Alexa Fluor 647 to visualize newly formed alpha synuclein aggregates). The mean raw image is compared to the second order 3D-SOFI without and with batching (batch size of 50 frames) on the raw data and trend-corrected data. The zoom of the area indicated in the full image showed in (**a**) is shown in (**b**) and reveals a better contrast, lower background signal and less intensity artefacts. All results were obtained using Matlab R2018b (Mathworks, USA).

As mentioned before, Whittaker smoothing is both computationally efficient and model-free, which makes it easier to apply the technique in a variety of settings. To illustrate this potential, we applied the method to three-dimensional SOFI data acquired using an image-splitting prism^33^. Figure 4 shows one color channel of a SOFI dataset containing both fluorescent proteins and organic dyes that have different photophysical behavior, acquired on a primary hippocampal neuron treated with alpha-synuclein pre-formed fibrils and antibody-labeled with Alexa Fluor 647 to visualize newly formed alpha synuclein aggregates (Methods section). Accumulation of this protein occurs in several neurodegenerative diseases, and is a hallmark for some of them, such as Parkinson’s disease. In addition to the mean image of the raw data, we show the results obtained with second order 3D-SOFI on the non-corrected data without and with batching (batches of 50 frames) and trend-corrected data (right panels). The 3D structures were visualized by color-coding the different planes and combining them using a maximum intensity z-projection (color bar indicated in the figure).

As Fig. 4 shows, the batching-corrected SOFI image shows a distinct lack of detail due to the high dynamic range of the image, with very bright features from perinuclear accumulation of long and thick alpha-synuclein fibers that mask lower-intensity zones of e.g., smaller alpha-synuclein species in the nucleus and thus not a lot of detail in the cellular structure can be observed. However, performing SOFI on the trend-corrected data results in images where more detail is apparent, which allows for a better investigation of these proteins.

## Conclusion

In this contribution, we have investigated the use of the model-free and computationally efficient Whittaker smoothing to correct SOFI images for slowly-varying distortions such as photodestruction. Whittaker smoothing is a global signal fitting procedure that adapts to local changes in the signal by using a penalty as a smoother. The result is the separation of signal contributions that appear at a lower timescale than typical for fluorophore blinking and the fluorophore blinking itself. We find that this procedure results in images that show more detail by eliminating the unspecific contribution of photodestruction, even when compared to the current state-of-the-art method batch correction, and can be applied on two- and three-dimensional data. Moreover, besides the visual improvements, removing the photodestruction is also essential to ensure the quantitative nature of SOFI in real-world samples. We expect that our approach will be generally useful in correcting for the presence of slow intensity variations in SOFI data and in other techniques that require stationary signals.

## Methods

### Whittaker smoothing

Whittaker smoothing, originally published in 1923^25^, was popularized in chemical applications by Eilers in 2003^26^, because it is a straightforward algorithm with many attractive properties. For a signal, **y**, of length *n* (sampled at equal distances), Whittaker smoothing can be reduced to linear regression in which the following least squares loss function is minimized.

1 $$S_{1} = {\text{min }}\left( {{\mathbf{||y}} - {{\varvec{\upmu ||}}}^{2} + { }\lambda ||{\mathbf{D\upmu|| }}^{2} } \right) .$$

In Eq. (1), the smooth series ${{\varvec{\upmu}}}$ is a fit to **Y** and the differencing matrix **D** is of order *d*. This loss function is divided into two parts, in which the least squares term assures a good fit, and the penalty term constrains the differences of the adjacent coefficients in ${{\varvec{\upmu}}}$, essentially reducing data variance and therefore having a smooth fit to the data. The weight of the penalty term, which is directly related to the smoothness of the fit, is controlled by the smoothing parameter $\lambda$ and can be automatically optimized using a V-curve optimization procedure^31,32^. Throughout this work, the differencing matrix is of order *d* = 2 as the best results were obtained with this order. For a signal of length *n* = 7, **D** is then given as

2 $${\mathbf{D}} = \left[ {\begin{array}{*{20}c} 1 & { - 2} & 1 & 0 & 0 & 0 & 0 \\ 0 & 1 & { - 2} & 1 & 0 & 0 & 0 \\ 0 & 0 & 1 & { - 2} & 1 & 0 & 0 \\ 0 & 0 & 0 & 1 & { - 2} & 1 & 0 \\ 0 & 0 & 0 & 0 & 1 & { - 2} & 1 \\ \end{array} } \right].$$

The solution to this problem is straightforward and is given as

3 $${\hat{\mathbf{\upmu }}} = \left( {{\mathbf{I}} + \lambda {\mathbf{D}}^{{\mathbf{T}}} {\mathbf{D}}} \right)^{ - 1} {\mathbf{y}},$$

in which **I** represents the identity matrix and ${\mathbf{D}}^{{\mathbf{T}}}$ is the transpose of **D**.

In our application, the measured imaging data of size ($x \times y \times n$) can be unfolded and transposed to a data matrix **Y**, of size ($n \times k$), where *n* is the number of acquired frames and *k* the number of pixels $(k = x \times y)$. This data matrix contains the pixel signals as column vectors that can be smoothed together. It is straightforward to rewrite Eq. (1) and its solution in Eq. (3) as

4 $$S_{2} = {\text{min }}\left( {{\mathbf{||Y}} - {\mathbf{M||}}^{2} + { }\lambda|| {\mathbf{DM||}}^{2} } \right) ,$$

5 $${\hat{\mathbf{M}}} = \left( {{\mathbf{I}} + \lambda {\mathbf{D}}^{{\mathbf{T}}} {\mathbf{D}}} \right)^{ - 1} {\mathbf{Y}},$$

where **M** is a matrix containing column vectors of smooth fits to the data **Y**. The other matrices in Eqs. (4) and (5) remain as explained above.

The smooth fit **M** to all the pixel signals in **Y** represents the photodestruction signal (and other smooth effects), while the trend-corrected data is obtained by rearranging the difference between the two matrices (i.e., **Y** − **M**) to the original 3D dimensions of the data. The procedure treats the data as a series of signals, meaning that all pixels are individually fit.

### Simulations

Simulations were used for the evaluation of correcting by Whittaker smoothing (and compared to other methods as in^24^). A stack of images with dimensions (32 *pixels* × 32 *pixels*) was simulated (500 frames) with a pixel size of 100 nm containing 20,000 fluorophores (on-time ratio of 9%; emission of 30 photons ms^-1^) located on 10 randomly oriented and intersecting lines. The fluorophores were simulated independently from all other fluorophores and were modelled as continuous-time Markov chains ($\tau_{on} = 10 \;{\text{ms}}$ and $\tau_{off} = 100 \;{\text{ms}}$) with the PSF simulated to be a 2D Gaussian (standard deviation determined by the numerical aperture (*NA* = 1.4) and wavelength ($\lambda = 520 \;{\text{nm}}$). The exposure time was set at 10 ms, and shot noise, a camera offset (1000), EM gain (50) and noise were included. Each pixel had a Poisson distributed background of 10 photons. The photodestruction was assumed to be distributed mono-exponentially and defined by the survival time. In these simulations, survival times $(\tau_{bl} )$ were set at 1.1 s, 5.5 s, 11 s and 33 s.

All simulations were repeated in 100-fold to have accurate statistics and were characterized by metrics such as signal-to-noise (SNR) and the root mean square deviation (RMSD). The SNR of the result is defined as the ratio between the value of the signal and the uncertainty of its estimate and is a measure for the precision of imaging (high SNR is better). The RMSD on the other hand allows to evaluate the accuracy of the correction method by quantitatively comparing the results to a reference SOFI image, which was obtained by averaging 100 SOFI images of large image sequences (5,000 images where no photodestruction was present). A low RMSD leads to more accurate images.

### Sample preparation

Transfection of the HeLa cells expressing Vimentin-Dreiklang was performed according to the protocol described in the work of Geissbuehler et al*.*^33^. In this protocol, 2 µL FuGENE 6 reagent (Promega) was incubated for 5 min in 33 µL Opti-MEM Reduced Serum Media (Life Technologies), to which 0.67 µg of the pMD-Vim-Dreiklang plasmid^34^ was added. After incubation, the solution was kept at room temperature for 30 min and then carefully added to the HeLa cells that were seeded in 1.5 cm^2^ wells. After addition, the cells were put back at 37 °C in the incubator and left overnight. Lastly, the medium was exchanged approximately 15–30 min before imaging to the antibleaching medium DMEM^gfp^-2 supplemented with rutin (Evrogen) to obtain a final concentration of 20 mg L^−1^.

The sample preparation of the primary hippocampal neuron–Alexa Fluor 647 immunolabelled α–synuclein aggregates was performed similar to the protocol described in the work of Descloux et al*.*^35^. Not considering the primary neuron culture preparation and the treatment of the hippocampal neurons with α-synuclein (α-syn) pre-formed fibrils, it works as follows. First, the neurons were washed twice with Phosphate Buffered Saline (PBS; Life Technologies) and then fixed in 4% paraformaldehyde at room temperature for twenty minutes. After fixation, the neurons were washed twice again with PBS and incubated in 3% Bovine Serum Albumin (BSA) in 0.1% Triton X-100 PBS (PBS-T) at room temperature for 30 min. The neurons were then incubated with the primary antibodies [1:2,000 dilution chicken anti-MAP2 (Abcam), 1:150 dilution mouse anti-α-tubulin (clone DM1α, Abcam) and 1:500 dilution rabbit anti-pS129-α-syn (MJFR-13, Abcam)] at room temperature for two hours. Cells were then washed five times with PBS-T and incubated subsequently with 1:400 dilution secondary donkey anti-chicken Alexa488 (Jackson ImmunoResearch), 1:100 dilution anti-mouse Janelia Fluor 549 (prepared using unlabeled antibody from Life Technologies) and 1:800 dilution anti-rabbit Alexa647 (Life Technologies). Before imaging, cells were washed with PBS-T (five times) and PBS (two times) and imaging buffer containing a thiol and an enzymatic oxygen scavenging system was applied (50 mM 2-Mercaptoethylamine in 50 mM Tris–HCl pH 8.0, 10 mM NaCl buffer containing 2.5 mM protcatechuic acid (PCA) and 50 nM Protocatechuate-3,4-Dioxygenase from Pseudomonas Sp. (PCD) with > 3 Units mg^−1^.

### Microscope setup

The microscope setup used is a custom-built system ^33^, equipped with an incubator to control temperature and CO_2_ for live-cell imaging, containing a 60 × water-immersion objective (1.2 NA; UPLSAPO 60XW, Olympus), two excitation and reactivation diode lasers (MLL-III-635, 800 mW, Roithner Lasertechnik and iBeam smart, 405 120 mW, Toptica), a green DPSS laser (MLL-FN-532, 800 mW, Roithner Lasertechnik) and an iXon DU 897 (Andor) EMCCD camera or a custom image splitting prism^35^ and two sCMOS cameras (ORCA Flash 4.0, Hamamatsu), depending on the application. Additionally, fluorescence light was filtered using a dichroic mirror (zt405/488/532/640/730rpc, Chroma) and an emission filter depending on the application as well.

For the two-dimensional application of the HeLa cells labelled with Dreiklang proteins, the former camera setup was used (at a framerate of 50 Hz; 5000 frames), in combination with the green DPSS (450 W cm^−2^, 532 nm) and iBeam diode (15 W cm^−2^; 405 nm) laser for tuning the blinking rate of the Dreiklang fluorescent protein (in addition to a 365 nm LED epi-illumination to tune the switching kinetics at 1.6 W cm^−2^). The emission filter used here was the Bright Line 582/75 (Semrock) filter.

In the three-dimensional application, the primary hippocampal neuron–Alexa Fluor 647 immunolabelled α–synuclein aggregates data was imaged with the latter setup (at a framerate of 50 Hz; 5000 frames) and the Roithner Lasertechnik (2 kW cm^−2^; 635 nm) and iBeam (7 W cm^−2^; 405 nm) diode lasers. The emission filter used here was the ZET405/488/532/640m (Chroma) filter.

## Supplementary Information

Supplementary Information

## Data Availability

The data used and analyzed in this work are available from the corresponding author upon reasonable request.

## References

[1] Hell SW, Wichman J (1994). Breaking the diffraction resolution limit by stimulated emission: stimulated-emission-depletion-microscopy. Opt. Lett..

[2] Rust MJ, Bates M, Zhuang X (2006). Sub-diffraction-limit imaging by stochastic optical reconstruction microscopy (STORM). Nat. Methods.

[3] Hess ST, Girirajan TPK, Mason MD (2006). Ultra-high resolution imaging by fluorescence photoactivation localization microscopy. Biophys. J..

[4] Betzig E (2006). Imaging intracellular fluorescent proteins at nanometer resolution. Science.

[5] Gustafsson MGL (2000). Surpassing the lateral resolution limit by a factor of two using structured illumination microscopy. J. Microsc..

[6] Muller CB, Enderlein J (2010). Image scanning microscopy. Phys. Rev. Lett..

[7] Vandenberg W, Leutenegger M, Lasser T, Hofkens J, Dedecker P (2015). Diffraction-unlimited imaging: from pretty pictures to hard numbers. Cell Tissue Res..

[8] Shannon CE (1949). Communication in the presence of noise. Proc. Inst. Radio Eng..

[9] Hugelier S (2016). Sparse deconvolution of high-density super-resolution images. Sci. Rep..

[10] Hugelier S, Eilers PHC, Devos O, Ruckebusch C (2017). Improved superresolution microscopy imaging by sparse deconvolution with an interframe penalty. J. Chemom..

[11] Nehme E, Weiss LE, Michaeli T, Shechtman Y (2018). Deep-STORM: super-resolution single-molecule microscopy by deep learning. Optica.

[12] Min J (2014). FALCON: fast and unbiased reconstruction of high-density super-resolution microscopy data. Sci. Rep..

[13] Babcock H, Sigal YM, Zhuang X (2012). A high-density 3D localization algorithm for stochastic optical reconstruction microscopy. Opt. Nanoscopy.

[14] Boyd N, Schiebinger G, Recht B (2017). The alternating descent conditional gradient method for sparse inverse problems. SIAM J. Optim..

[15] Hebert S, Costantino PW, Wiseman P (2005). Spatiotemporal image correlation spectroscopy (STICS) theory, verification, and application to protein velocity mapping in living CHO cells. Biophys. J..

[16] Ruckebusch C (2015). Mapping pixel dissimilarity in wide-field super-resolution fluorescence microscopy. Anal. Chem..

[17] Dertinger T, Colyer R, Lyer G, Weiss S, Enderlein J (2009). Fast, background-free, 3D super-resolution optical fluctuation imaging (SOFI). Proc. Natl. Acad. Sci. USA.

[18] Sroda A (2020). SOFISM: super-resolution optical fluctuation image scanning microscopy. Optica.

[19] Dertinger T, Colyer R, Vogel R, Enderlein J, Weiss S (2010). Achieving increased resolution and more pixels with superresolution optical fluctuation imaging (SOFI). Opt. Express..

[20] Dertinger T, Xu J, Foroutan-Naini O, Vogel R, Weiss S (2012). SOFI-based 3D superresolution sectioning with a widefield microscope. Opt. Nanoscopy.

[21] Vandenberg W, Leutenegger M, Duwé S, Dedecker P (2019). An extended quantitative model for super-resolution optical fluctuation imaging (SOFI). Opt. Express.

[22] Vandenberg W (2016). Model-free uncertainty estimation in stochastical optical fluctuation imaging (SOFI) leads to a doubled temporal resolution. Biomed. Opt. Express.

[23] Vandenberg W, Dedecker P (2017). Effect of probe diffusion on the SOFI imaging accuracy. Sci. Rep..

[24] Peeters Y (2017). Correcting for photodestruction in super-resolution optical fluctuation imaging. Sci. Rep..

[25] Whittaker ET (1923). On a new method of graduation. Proc. Edinb. Math. Soc..

[26] Eilers PHC (2003). A perfect smoother. Anal. Chem..

[27] Cobas C (2018). Applications of the Whittaker smoother in NMR spectroscopy. Magn. Reason. Chem..

[28] Eilers PHC (2003). Parametric time warping. Anal. Chem..

[29] Zhang ZM, Chen S, Liang YZ (2010). Baseline correction using adaptive iteratively reweighted penalized least squares. Analyst.

[30] Chountasis S, Katsikis VN, Pappas D, Perperoglou A (2012). The Whittaker smoother and the Moore-Penrose inverse in signal reconstruction. Appl. Math. Sci..

[31] Frasso G, Eilers PHC (2015). L- and V-curves for optimal smoothing. Stat. Model..

[32] Frasso, G., Eilers, P.H.C. Smoothing parameter selection using the L-curve. Technical report, Erasmus Medical Center, Erasmus Universiteit, Rotterdam, The Netherlands, (2012).

[33] Geissbuehler S (2014). Live-cell multiplane three-dimensional super-resolution optical fluctuation imaging. Nat. Commun..

[34] Brakemann T (2011). A reversibly photoswitchable GFP-like protein with fluorescence excitation decoupled from switching. Nat. Biotechnol..

[35] Descloux A (2018). Combined multi-plane phase retrieval and super-resolution optical fluctuation imaging for 4D cell microscopy. Nat. Phot..

