# Supplementary material for: Smoothness correction for better SOFI imaging
Source: Sci Rep. 2021 Apr 7;11:7569. doi: 10.1038/s41598-021-87164-4 (PMC8027426; doi:10.1038/s41598-021-87164-4)
Supplement: Supplementary file 1 — Supplementary Information [file 41598_2021_87164_MOESM1_ESM.pdf]

Supplementary Information: Smoothness correction for better SOFI imaging

Siewert Hugelier[1,*], Wim Vandenberg[1], Tomáš Lukeš[2], Kristin S. Grußmayer[2,3], Paul H.C. Eilers[4], Peter Dedecker[1] and Cyril Ruckebusch[5]

[1]Laboratory for Nanobiology, KU Leuven, B-3001 Leuven, Belgium.

[2]Laboratory of Nanoscale Biology, École Polytechnique Fédérale de Lausanne, 1015 Lausanne, Switzerland.

[3]Grußmayer Lab, Delft University of Technology, 2629 HZ Delft, the Netherlands.

[4]Erasmus University Medical Centre, 3015 Rotterdam, the Netherlands.

[5]Univ. Lille, CNRS, UMR 8516, LASIRE, F-59000 Lille, France.

[*]Correspondence should be addressed to Siewert Hugelier (e-mail: siewert.hugelier@gmail.com).

Supplementary Notes 1	2
Supplementary Figure 1	4
Supplementary Figure 2	6
Supplementary References	7

Supplementary Notes 1

A V-curve procedure [1], [2] can be used to determine the smoothing parameter, λ, automatically. We recall the minimization problem of Whittaker smoothing [3], [4] and its solution in equation (SI1) and equation (SI2), respectively

$$S = \min\left(\|\mathbf{Y} - \mathbf{M}\|^2 + \lambda\|\mathbf{DM}\|^2\right) \quad \text{Eq. (SI1)}$$

$$\widehat{\mathbf{M}} = \left(\mathbf{I} + \lambda\mathbf{D}^{\mathbf{T}}\mathbf{D}\right)^{-\mathbf{1}}\mathbf{Y} \quad \text{Eq. (SI2)}$$

with $\mathbf{Y}$ being the matrix containing the pixel signals as column vectors of size $(n \times k)$ (with n being the length of the image sequence in frames and k the number of pixels), $\mathbf{M}$ the matrix containing the smooth fits to these signals and $\mathbf{D}$ the differencing matrix of order 2. $\mathbf{I}$ is the identity matrix and $\mathbf{D}^{\mathbf{T}}$ is the transpose of the differencing matrix $\mathbf{D}$.

The V-curve is derived from the L-curve [5], a parametric plot of $\phi(\lambda)$ vs $\psi(\lambda)$, defined as

$$\phi(\lambda) = \log_{10}\|\mathbf{Y} - \mathbf{M}\|^2 \quad \text{Eq. (SI3)}$$

$$\psi(\lambda) = \log_{10}\|\mathbf{DM}\|^2 \quad \text{Eq. (SI4)}$$

in which the optimal value can be found by computing the curvature of the curve. However, computations for this are not easy as it requires calculating first and second derivatives of $\phi(\lambda)$ and $\psi(\lambda)$, but, when the L-curve shows a distinguishable convex area, an alternative approach called the V-curve can be used. As described in the work of Frasso and Eilers [1], the maximum curvature of the L-curve can be approximated by simply calculating the Euclidean distance in the (ψ, ϕ) space. This is because the size of $\Delta\psi$ and $\Delta\phi$ depend on the size of $\Delta\log_{10}(\lambda)$ and going towards the limits means calculating the derivative of the path length with respect to $\log_{10}(\lambda)$. The optimal smoothing parameter is then chosen by minimizing the following

$$\min\left\{\sqrt{(\Delta\phi)^2 + (\Delta\psi)^2}\right\}. \quad \text{Eq. (SI5)}$$

In the procedure, Eq. (SI5) is calculated for each pixel trace simultaneously (but independently). However, in this work, we opted to optimize the smoothing in a global way, to avoid

issues with spatial correlation between the different pixels, by averaging over all pixels, as the values obtained for λ ranged between 8.92×10^7 and 2.79×10^9, and led to results that were almost identical.

For the data at hand, the result of this procedure is shown in Supplementary Figure 1, where a set of λ between 10^{-3} – 10^{14} was investigated. We show the mean image of the raw data over 5,000 frames in Supplementary Figure 1a and the evolution of the step size vs the smoothing parameter (V-curve) in Supplementary Figure 1b. There is a clear minimum present in this curve which is indicated by the green line. Supplementary Figure 1c then shows the corrected images for the three selected λ values as indicated in Supplementary Figure 1b ($\lambda_1 = 3.73$; $\lambda_2 = 2.96 \times 10^8$ and $\lambda_3 = 2.47 \times 10^{11}$, respectively). From these panels, we see that the mean corrected image at the optimal value for λ (i.e. point 2) shows the best image contrast and the most details can be noticed. To confirm this point, raw signals of two pixels (grey curves) are shown for the three situations (Suppl. Fig. 1d), their fits (red curves), and the trend-corrected signal (green curves). In the top and bottom panel (the non-optimal situations), one can notice that either high frequency signal, containing information useful for SOFI, was being fitted as well (and was thus be removed from the trend-corrected data), or the general photodestruction trend was not being fitted (creating artefacts in the process), respectively. Of the three situations, only when the optimal λ value was used (i.e. situation 2), a reasonable smooth signal was being fitted and we can claim that the trend-corrected pixels signals were representative for the blinking of our probes. The time needed to complete the automatic estimation of λ using the V-curve procedure on this data was roughly 600 seconds (for 1,500 tested λ values) on an Intel(R) Core i7-8750H CPU @ 2.20 GHz. Note that in this work the λ range was larger than what usually would be investigated to showcase the influence of λ.

Supplementary Figure 1

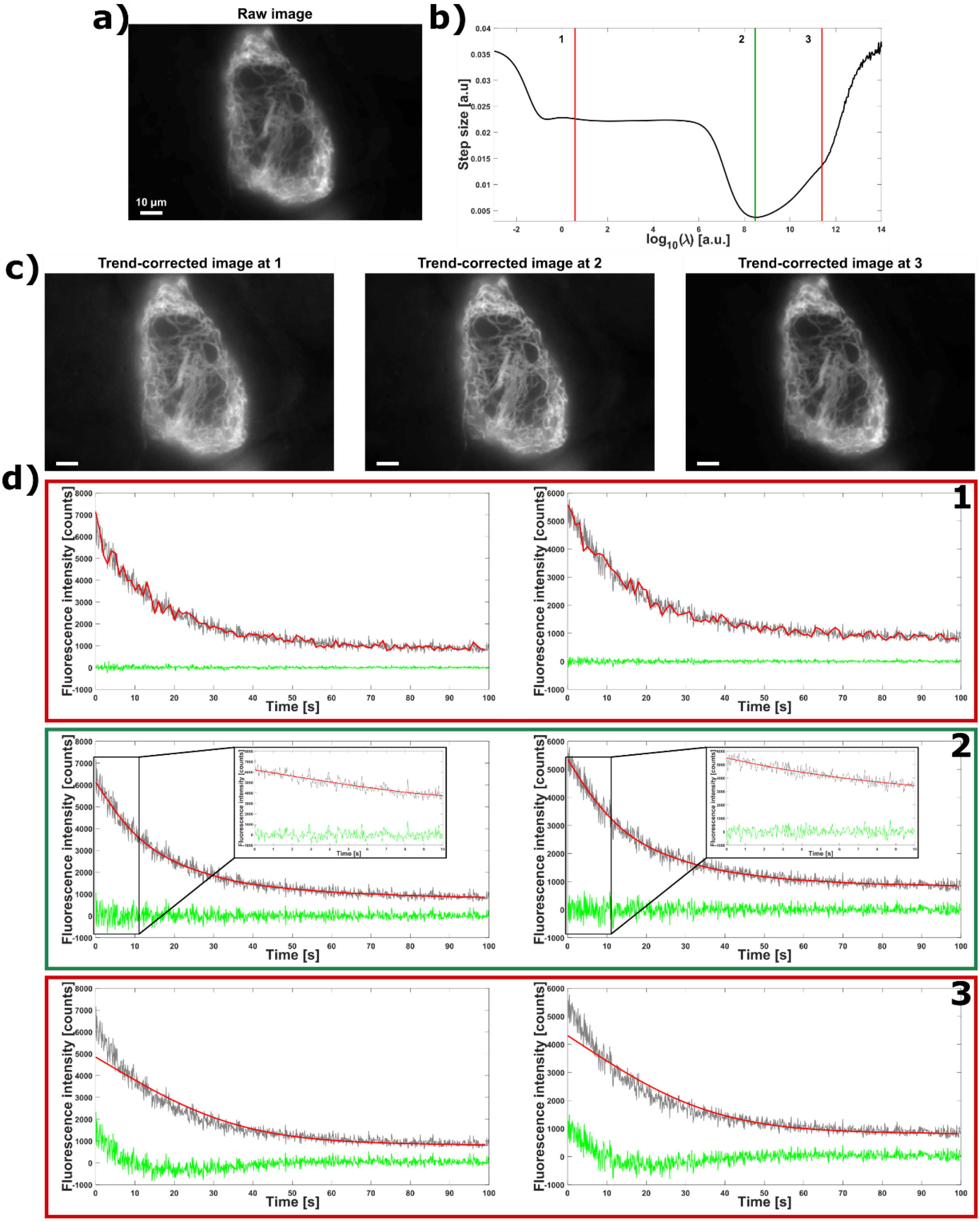

Supplementary Figure 1 A showcase of the V-curve method to automatically select the smoothing parameter on the entire data set. The mean raw image of the data is shown in (a), whereas (b) shows the plot of the step size vs the smoothing parameter (V-curve). Three different mean corrected images

are shown in (c) for a situation where λ is too low, optimal and too high, as indicated by the lines in (b) ($\lambda_1 = 3.73$; $\lambda_2 = 2.96 \times 10^8$ and $\lambda_3 = 2.47 \times 10^{11}$, respectively). The effect of Whittaker smoothing on two pixels for the three situations is shown in (d), with the middle panel being the optimally smoothed signals (grey: raw pixel trace; red: fitted trace; green: trend-corrected pixel trace). All results were obtained using Matlab R2018b (Mathworks, USA).

Supplementary Figure 2

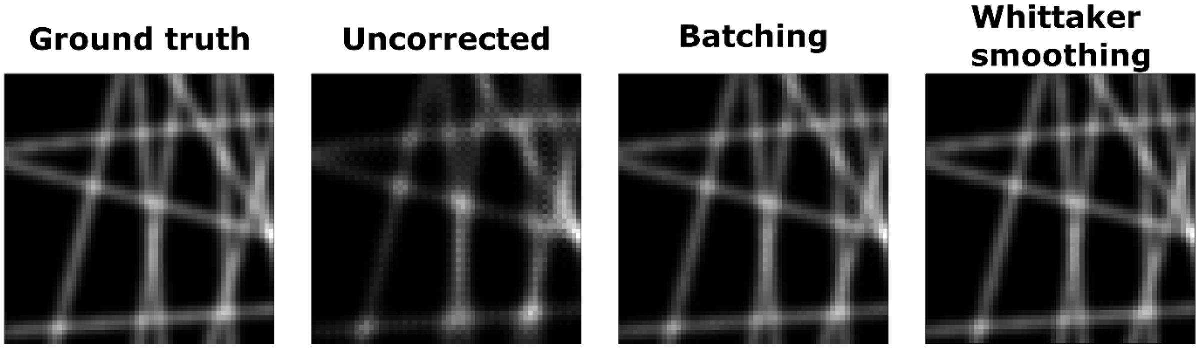

Figure 2 Results obtained on simulated data with fast photodestruction (survival time: $\tau_{bl} = 1.1\ s$). We show the comparison between the ground truth SOFI image, the SOFI image obtained from uncorrected data, the SOFI image obtained from batched data and the SOFI image obtained from trend-corrected data. All results were obtained using Matlab R2018b (Mathworks, USA).

Supplementary references

[1] Frasso, G., Eilers, P.H.C. L- and V-curves for optimal smoothing. *Stat. Model.* **15**, 91–111 (2015).

[2] Frasso, G., Eilers, P.H.C. Smoothing parameter selection using the L-curve. Technical report, Erasmus Medical Center, Erasmus Universiteit, Rotterdam, The Netherlands, 2012.

[3] Whittaker, E.T. On a New Method of Graduation. *Proc. Edinb. Math. Soc.* **41**, 63–75 (1923).

[4] Eilers, P. H. C. A perfect smoother. *Anal. Chem.* **75**, 3631–3636 (2003).

[5] Hansen, P.C. Analysis of discrete ill-posed problems by means of the L-curve. *Siam Review* **34**, 561–580 (1992).
